# A comprehensive review: Impact of oleogel application on food texture and sensory properties

**DOI:** 10.1002/fsn3.4110

**Published:** 2024-04-13

**Authors:** Lingyi Liu, Zengli Gao, Gang Chen, Jiaying Yao, Xinyu Zhang, Xiaoting Qiu, Lianliang Liu

**Affiliations:** ^1^ State Key Laboratory for Managing Biotic and Chemical Threats to the Quality and Safety of Agro‐Products, Key Laboratory of Animal Protein Deep Processing Technology of Zhejiang, Zhejiang‐Malaysia Joint Research Laboratory for Agricultural Product Processing and Nutrition, School of Food and Pharmaceutical Sciences Ningbo University Ningbo Zhejiang China; ^2^ Department of Food Science and Technology University of Nebraska‐Lincoln Lincoln Nebraska USA; ^3^ Inner Mongolia Enterprise Key Laboratory of Dairy Nutrition Health & Safety, Inner Mongolia Mengniu Dairy (Group) Co., Ltd. Huhhot China

**Keywords:** oleogel, oleogel‐based food, saturated fat replacer, sensory, texture

## Abstract

Oleogels, characterized by their semisolid matrix formed from liquid oil structured by gelators, are emerging as a pivotal innovation in food formulation, primarily due to their capacity to enhance the nutritional profile of products by incorporating healthier fats. This review explored the integration of oleogels into diverse food matrices, examining their impact on texture, mouthfeel, and overall sensory characteristics. Through an extensive analysis of current research, this paper illustrates the versatility of oleogels created with a variety of structuring agents across different food applications. It also addresses the challenges inherent in the use of oleogels, including the preservation of their stability and consistency through varying storage and processing conditions, navigating the regulatory landscape concerning oleogelator safety and acceptability, and confronting higher production costs. Overall, this comprehensive review highlights the potential of oleogels as a promising tool for achieving desirable textural and sensory attributes in food products while also identifying areas for future research and development.

## Graphical abstract

This review highlights the application of oleogels in food innovation, focusing on their ability to improve nutritional value and impact texture and sensory properties. It covers the oleogel application across various food products, their formulation challenges, and the need for further research to optimize their use and consumer acceptance.

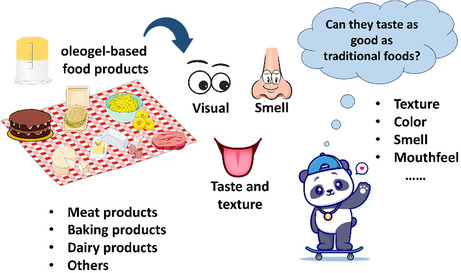

## 1 INTRODUCTION

Solid fats play a pivotal role in achieving the desired textural and sensory characteristics in various food products. For instance, solid fats are essential in achieving the desired crumb structure and mouthfeel in baked foods (Blake & Marangoni, 2015). In the context of ice cream, saturated fats are crucial for carrying and stabilizing flavors and aromas, contributing to the richness and creaminess of the product (Afoakwa, 2016). However, the high consumption of solid fats, which are rich in saturated fatty acids (SFAs) or trans fatty acids (TFAs), has been associated with an elevated risk of various health issues, including obesity, coronary heart disease, cancer, diabetes, and allergies (Liu et al., 2017). Although the elimination of TFAs from diets can be achieved by avoiding hydrogenated fats in food formulations, saturated fat remains the predominant lipid present in most processed foods. Given the growing consumer demand for healthier food options, there has been a surge in research aimed at reducing saturated fat content while maintaining the desirable texture profiles and sensory attributes of food products. In this pursuit, the development of solid‐like oleogels from unsaturated vegetable oils has recently emerged as a promising healthier alternative to traditional solid fats.

Oleogels are structured lipid systems in which liquid oil is combined with a gelling or structuring agent, resulting in a semisolid or gel‐like state (Tan et al., 2023). The transformation from liquid oil to a more solid form has captured attention due to the growing preference for healthier food choices since this change is achieved without modifying the fatty acid profile of the oil (Dominguez et al., 2024). Additionally, oleogels are gaining increasing recognition in the food industry for their ability to encapsulate and deliver bioactive compounds, such as antioxidants and flavoring agents. This characteristic not only provides solutions to various food production challenges but also paves the way for the development of novel food products (Pinto et al., 2021). The transformation of oils into a semisolid form imparts distinctive textural qualities, offering new avenues for enhancing the sensory appeal of food products. By maintaining the desired texture and stability while incorporating liquid oils, oleogels present a promising approach for improving the sensory experience of a wide range of food products (Issara, 2022).

Texture and sensory attributes are crucial in food product development and consumer acceptance (Garvey et al., 2020). The texture, consistency, mouth‐coating properties, and structural integrity of food products are influenced by various factors such as composition, structure, moisture content, particle size, and rheological properties. Similarly, sensory attributes, including flavor release, aftertaste, and tactile sensation, significantly impact how consumers perceive the quality, freshness, and desirability of a food product (Abdel‐Moemin et al., 2018). These elements not only affect the immediate sensory experience but can also evoke emotions and memories, thereby shaping consumer perceptions of quality and freshness. Oleogels have emerged as a promising alternative food formulation, effectively replacing solid fats without compromising the sensory properties or functionality of the end product. Aligning with recommendations to reduce saturated fat intake and eliminate trans fats from the diet, oleogels provide a valuable solution to create healthier food options. Multiple comprehensive reviews and book chapters have focused on oleogel developments, mechanisms, properties, health implications, and potential food applications (Feichtinger & Scholten, 2020; Gutiérrez‐Luna et al., 2022; Kavya et al., 2014; Silva et al., 2022; Tan et al., 2023; Wang et al., 2022). However, there remains a gap in systematic reviews addressing the specific impact of oleogel formation on the textural and sensory qualities of food products.

Therefore, this paper aims to bridge this gap by comprehensively exploring the transformative effects of oleogels on textural and sensory characteristics in various food applications. This comprehensive exploration is vital for understanding how oleogels can enhance the complexity and appeal of food textures and flavors, aligning with the evolving preferences and health consciousness of modern consumers.

## 2 UNDERSTANDING OLEOGEL FORMATION

Oleogels are semisolid systems with viscoelastic properties and a hydrophobic nature, achieved by the process of organogelation (Guo et al., 2023; Huang et al., 2023; Tan et al., 2023). This unique characteristic gives oleogels their gel‐like texture while still maintaining the fluidity and spreadability of oil. The formation of oleogels is facilitated by intricate molecular interactions, which effectively convert liquid oils into structured, semisolid, or gel‐like substances. These molecular interactions can be governed by both direct and indirect methods (Gengatharan et al., 2023).

### 2.1 Direct methods

**FIGURE 1:**
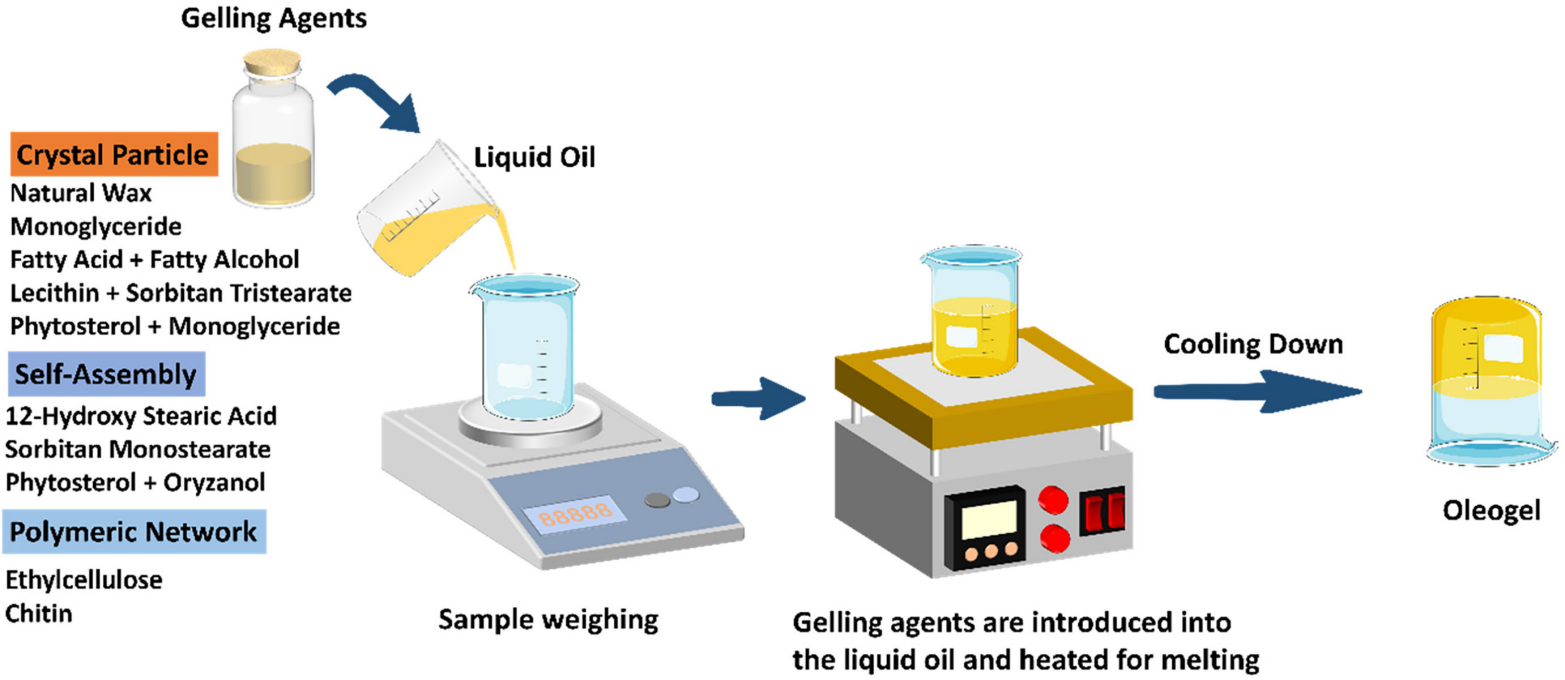
Direct method for oleogel preparation.

Direct methods in oleogelation involve dispersing molten oleogelators into an oil medium, followed by a cooling step to form a self‐supporting gel. This approach is recognized for its simplicity in oleogel production. The process starts with the introduction of oleogelators into the liquid oil, followed by cooling to initiate nucleation (Martins et al., 2020). This nucleation then leads to crystal growth and the subsequent stabilization of the crystal lattice. Ultimately, this results in forming a three‐dimensional network that entraps the liquid oil phase, forming an oleogel (Figure 1).

This process can be achieved using various gelling agents, mainly categorized into crystal particles, self‐assembly, and natural polymers (Wang et al., 2022). Crystal particles such as monoglyceride, fatty acids/fatty alcohols, phytosterol, and natural waxes are commonly employed as oleogelators (Wang et al., 2022). These agents function similar to high‐melting‐point solid fats, creating a structure that restricts the mobility of liquid oil, thus imparting gel‐like characteristics to the resultant product. However, the crystal structures developed by these oleogelators exhibit a morphology that is distinctly different from those seen in conventional solid fats. For instance, beeswax forms a less dense, fibrous network, leading to an oleogel with a semisolid structure (Doan et al., 2017). This structure is typically softer and more spreadable compared to the more rigid and compact nature of traditional solid fats. Additionally, oleogelators such as 12‐hydroxy stearic acid, sorbitan monostearate, and mixtures of γ‐oryzanol and β‐sitosterol, among others, demonstrate the capacity for self‐assembly. This process involves the initial structural units experiencing one‐dimensional growth, helix formation, and torsion within the oil phase. Among all polymer oleogelators, ethylcellulose and chitin have been reported to structure oil directly (Milani & Naeli, 2020; Shao et al., 2023). When ethylcellulose is dissolved in an oil medium and heated, it forms a homogeneous solution. Upon cooling, ethylcellulose molecules aggregate and create a three‐dimensional network structure through physical entanglements and possibly some secondary bonding. The ethoxy groups in ethylcellulose contribute to its solubility in oil and play a role in the gelation process. The resulting oleogel typically has a structure resembling a coral‐like polymer network with numerous pockets or holes. Chitin is a natural polysaccharide that forms through the linear arrangement of N‐acetylglucosamine units. In oleogel formation, chitin undergoes a dissolution process in specific solvents or under certain conditions and then forms a gel network when reintroduced into an oil medium. The gelation mechanism involves the reassembly of chitin molecules into a three‐dimensional network, primarily through hydrogen bonding and hydrophobic interactions. The structure of chitin‐based oleogels can vary depending on the processing conditions and the degree of deacetylation of chitin (Gallego et al., 2013).

### 2.2 Indirect methods

**FIGURE 2:**
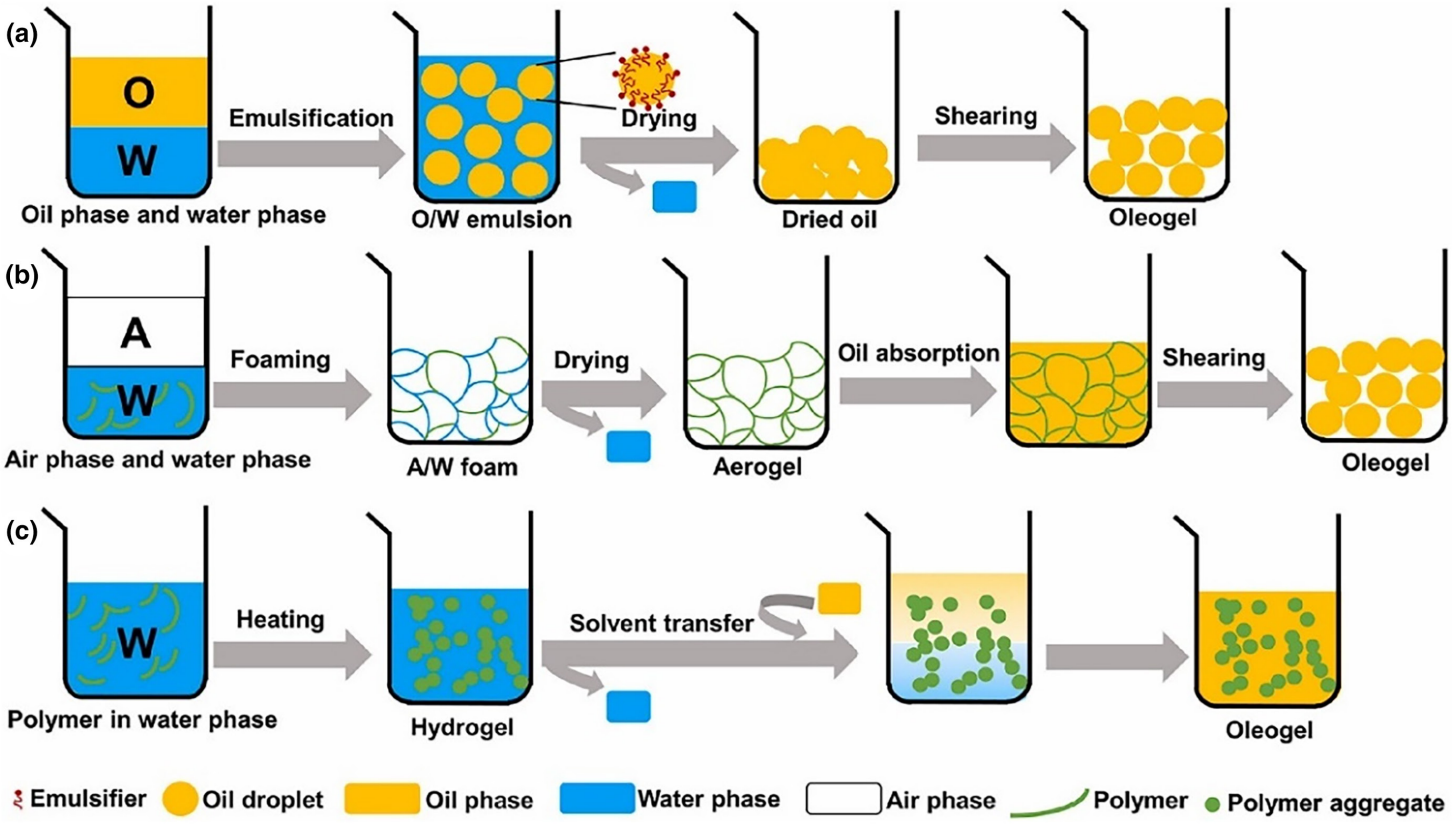
Indirect method for oleogel preparation (Guo et al., 2023). (a) Emulsion‐templated method; (b) form‐templated method; (c) solvent exchange method.

Indirect methods for oleogel preparation have emerged as a compelling alternative to direct methods, which typically depend on gelling agents that are sensitive to temperature and shear forces and often necessitate relatively high concentrations for effective oleogelation (Li et al., 2022). Indirect methods, such as utilizing abundant and low‐cost biopolymers, offer a more economical and broadly applicable approach for oleogel production. Indirect approaches involve using hydrocolloids or hydrophilic biopolymeric oleogelators as the primary building blocks for oleogels. This method prepares oleogels by exchanging water with a lipid continuous phase. Common indirect approaches for oil structuring include biphasic emulsion (i.e., emulsion template, foam template, and high internal phase emulsion) and solvent exchange where a structural framework is formed in a solvent comprising water or in a water‐based emulsion (Figure 2; Alavi & Ciftci, 2022; Guo et al., 2023; Li et al., 2022).

In the case of biphasic emulsions, food biopolymers like proteins, polysaccharides, sugar alcohols (including sorbitol derivatives), cellulose, polyphenol, and chitin complexes are initially dispersed within the aqueous phase to create an oil‐in‐water emulsion (Tan et al., 2023). Surface‐active polymers are pivotal in establishing a structural framework within this oil‐in‐water emulsion. Upon subsequent water removal through freeze‐drying, a gel network forms where oil droplets become densely packed within the polymer matrix (Qiu et al., 2018). This polymer network is then subjected to shearing forces to yield an oleogel characterized by tightly packed oil droplets enveloped by the polymer, which are evenly dispersed or clustered as isolated droplets within the continuous phase. Nevertheless, the biphasic emulsion method comes with inherent limitations in oleogel formation. The removal of the aqueous phase during the freeze‐drying process of hydrogels may result in internal collapse of the polymer network due to the heightened internal pressure generated by the formation of small crystals before drying (García‐González et al., 2012).

The solvent exchange approach offers a solution to mitigate the pore collapse phenomenon often observed in biphasic emulsion templates (Manzocco et al., 2017). This method uses alcohol or acetone to substitute the aqueous phase within the hydrogels. Subsequently, the organic solvents are removed using supercritical carbon dioxide to produce aerogels. Following, the oil phase is introduced into the polymeric network of the aerogels through a series of dipping or immersion processes, during which the aerogels absorb the oil to create oleogels.

## 3 IMPACT OF OLEOGELS ON THE TEXTURE AND SENSORY PROPERTIES OF FOOD PRODUCTS

**FIGURE 3:**
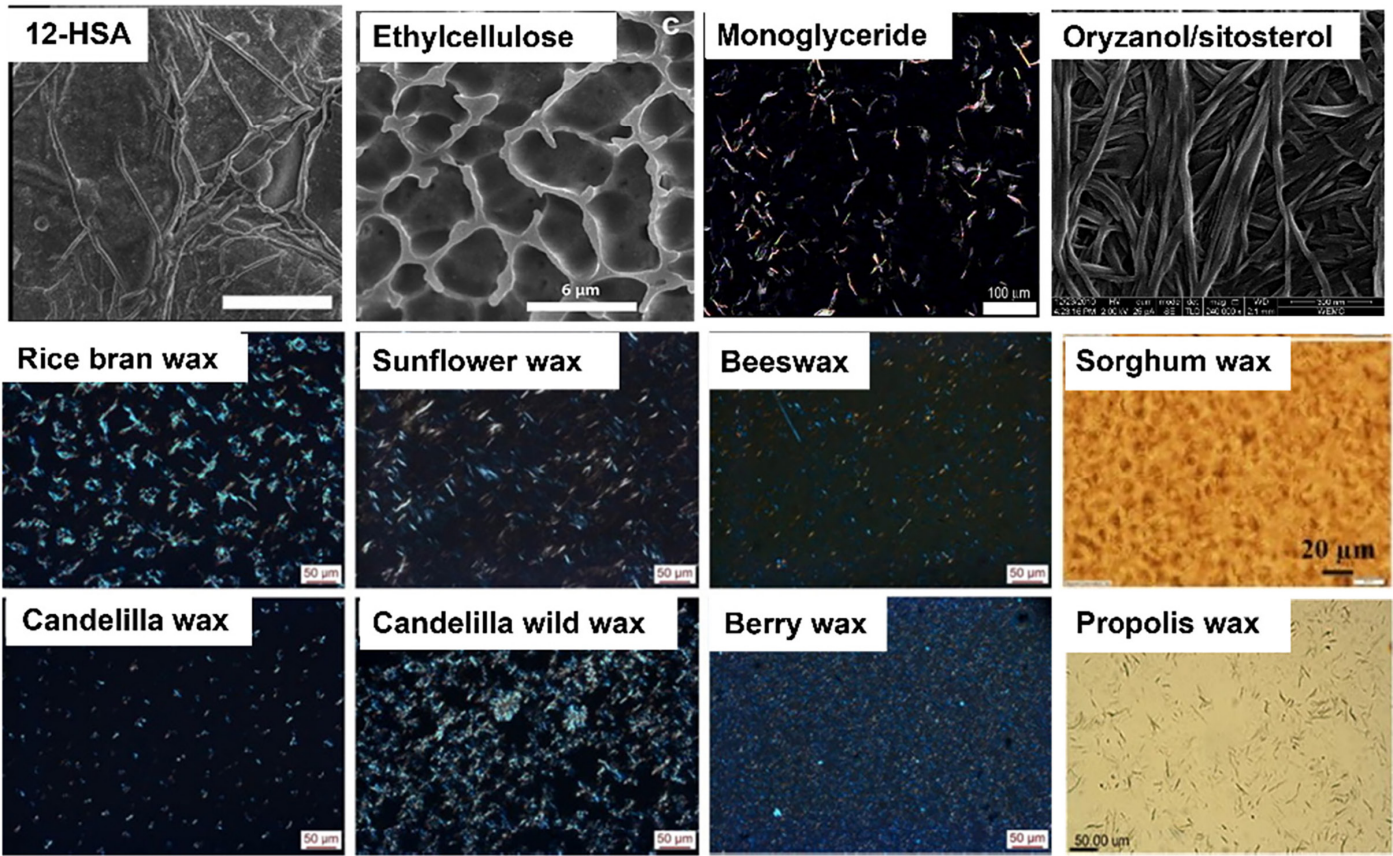
Crystal morphology of oleogelators. (Doan et al., 2017; Fayaz et al., 2017; Lopez‐Martinez et al., 2014; Rogers et al., 2009; Sawalha et al., 2013; Zetzl et al., 2014).

The exploration of oleogel applications and their consequential impact on food texture is of paramount importance for the advancement of healthier food alternatives. Oleogels, characterized by their variable physical properties contingent upon composition and processing conditions, present both a challenge and an opportunity to mimic the textural attributes of conventional solid fats. While some studies successfully achieved the desired texture, not all demonstrated texture attributes were satisfactory when compared to controls using saturated fats. For instance, the use of 15% carnauba wax in frying snacks resulted in a perceivable waxy taste (Chauhan et al., 2022). The gel network established by gelling agents plays a critical role in providing structural support. It is important to note that different sources of gelling agents may yield contradictory results. As shown in Figure 3, the microstructural properties of oleogels vary considerably based on the type of oleogelator used, which in turn can markedly affect the physical attributes of the foods incorporating these oleogels. This variability underscores the importance of selecting appropriate gelling agents to achieve the desired textural and structural properties in food products. Also, when incorporating oleogels into food products, it is crucial to consider the potential changes in their physical properties that may occur due to various factors, including the oil medium, processing conditions, and environmental influences. For example, Oil viscosity and polarity appear to have a significant impact on the gelation kinetics and crystallization behavior of oleogels. It has been found that the interaction between the gelator molecule and oil in ricinoleic acid‐based oleogels was improved as oil polarity increased, resulting in a decrease in gel strength (Mahmud et al., 2023). In addition, the van der Waals force in diacylglycerol oil oleogels was found stronger than the hydrogen bonding interactions induced by ethylcellulose and monoglyceride in triacylglycerol oil (Qiu et al., 2022).

Texture is a crucial factor in shaping consumer preferences for food products, and oleogels offer a valuable means of tailoring texture with their versatile structural properties. By substituting solid fats with oleogels, it becomes possible to achieve the desired textural attributes that enhance mouthfeel and stability (Badem & Baştürk, 2023; Espert et al., 2023). The semisolid nature of oleogels imparts a smooth and silky sensation, mimicking the creamy mouthfeel associated with high‐fat foods. Their structured composition enhances richness and indulgence perception, elevating the overall sensory experience. Additionally, oleogels can prolong flavor release, enhancing the overall taste perception. However, summarizing the intricate impact of oleogels on food product texture remains challenging due to the complexity of multiple factors. The specific food matrix, including different ingredients and processing methods, can also result in varying interactions during the manufacturing process and storage periods. This exploration is critical not only for achieving the primary objective of reducing unhealthy saturated and trans fats in diets but also for maintaining consumer acceptance through consistent texture and mouthfeel. It is crucial to acknowledge that the food matrix is a complex system, and the outcomes can vary significantly depending on the specific characteristics of different food systems.

### 3.1 Meat products

The processed meat product industry has been showing a full bloom and is witnessing an increasing trend in consumption due to worldwide popularity. Among the approaches directed to replace or reduce the usage of saturated fats, the oleogel application showed unique benefits based on nutrition and environmental sustainability. Table 1 shows that developing healthy meat products using oleogels involves sausages, patties, burgers, luncheon meat, and some local meat products. The role of fat in shaping sensory perceptions is undeniable. Composed of saturated fatty acids, fat tissues undergo significant phase transitions during cooking and mastication. As the temperature rises during cooking or when subjected to the warmth of the mouth during consumption, a portion of the fat undergoes melting, transitioning into a fluid state. Simultaneously, fat‐soluble compounds previously encapsulated within the fat structure are liberated. This dual process results in the creation of what is commonly referred to as meat juice, as it blends with the moisture naturally released during cooking. The presence of juiciness is pivotal in defining the sensory experience of meat products. Without it, meat products tend to take on a dry, tough, and rough texture rather than the desired tenderness and succulence.

**TABLE 1:** Effect of oleogel applications on the texture and sensory properties of meat products.

Food matrix	Oleogelator	Oil medium	Texture impacts	Sensory impacts	References
Sausage	Ethylcellulose (6%, 8%, 10%, 12%)	Sunflower oil, peanut oil, corn oil, flaxseed oil	Increased gelator caused higher brightness and hardness; Hardness decreased with higher oleogel substitution	Color and odor scores increased; 10%–30% peanut oil oleogel replacement was acceptable	Shao et al. (2023)
	Monoglyceride: beeswax 2:1 (20%)	Sunflower oil	10% oleogel replacement reduced texture and juiciness	7% oleogel replacement did not deteriorate the organoleptic properties	Igenbayev et al. (2023)
	Monoglyceride (5%)	High oleic sunflower oil, sunflower oil	50% replacement was comparable to control; Less resilient and cohesiveness with 100% replacement	Overall acceptable; less pink color	Ferro et al. (2021)
Meat patties	Rice bran wax (5%, 7%), candelilla wax (3%, 7%)	Hemp oil	7% rice bran wax sample comparable to control; Candelilla wax decreased the intensity of meaty odor and taste	Odor scores lower than control Reduced juicer value and softness	Hamidioglu et al. (2022)
Sucuk	Beeswax (10%), sunflower wax (10%)	Flaxseed oil	Textural values were lower	Lower sensory scores and consumer acceptance	Yilmaz and Toksoz (2022)
Meat analogs	Whey protein isolate emulsion (17%)	Soybean oil	Enhanced oil release	Improved fat‐related sensory attributes	Han et al. (2023)
Luncheon meat	Soy protein isolate (5%), highland barley β‐glucan	Linseed oil	Reduced the hardness	Decreased acceptance with increased oleogel substitution, 25% substitution is acceptable	Ma et al. (2023)

The replacement of traditional fats with oleogels can bring about improvements in the lipid profile of meat products. However, the impact on texture and sensory attributes largely depends on the mechanism of gelation employed. The direct oleogelation method, where the gelling agent is mixed directly into the liquid oil to prepare the oleogel, can sometimes result in an uneven distribution of fat throughout the product, leading to inconsistencies in texture and mouthfeel. For example, recent research findings shed light on the effects of wax‐based oleogels in meat products. These oleogels have been shown to reduce hardness, cohesiveness, gumminess, and chewiness values in products like sucuk (Yilmaz & Toksoz, 2022). In another study by Hamidioglu et al. (2022), wax‐based oleogels were found to match the texture properties of pork fat, such as spreadability and adhesiveness, in meat patties. However, they also resulted in a decrease in juiciness and taste intensity when compared to meat patties containing 100% pork fat. The concentration of oleogel used in the direct oleogelation method significantly influences the final texture of meat products (Ghiasi & Golmakani, 2022; Oh et al., 2019). Those research findings suggest that the oleogel‐based products received lower scores for overall acceptability than the control, underscoring the need for increased efforts to better meet consumer demands.

During cooking, the high temperature can break the bonds within the oleogel structure, leading to an unstable network. The presence of polymers such as proteins and polysaccharides can strengthen hydrogen bonding and improve tenderness, juiciness, and overall sensory acceptance of meat products. For instance, da Silva et al. (2019) developed an oleogel with pork skin, water, and high oleic sunflower oil, which significantly reduced cooking loss and increased emulsion stability when replacing backfat in Bologna‐type sausages. The probable reason is that Pork skin collagen, interacting with proteins, developed a more rigid gel matrix, preventing water and fat exudation during cooking (Wolfer et al., 2018). Since the polymer has been used in the indirect method for oleogel preparation, recent interest has also focused on investigating the effects of oleogels prepared using indirect methods. Meat products prepared with oleogels using indirect methods have shown higher customer satisfaction. For example, Han et al. mimicked juiciness in meat products through an oleogel, where vegetable oil was structured by a network of whey protein isolates (WPI) and water (Han et al., 2023). They found that adding oleogel significantly enhanced oil release and improved fat‐related sensory attributes in meat analogs. Another study involved a linseed oil‐based oleogel produced using an indirect method that incorporated soy protein isolate and highland barley β‐glucan (HBG) via an emulsion‐templated approach and this oleogel served as a healthier replacement for solid fats in the manufacturing of luncheon meat (Ma et al., 2023). Sensory evaluation results indicated that substituting 25% of the pork backfat with oleogel resulted in the desired overall acceptance level. In addition, the odor effect is also one concern in meat application.

### 3.2 Baking products

Oleogels have been recognized as an innovative approach to substitute or minimize traditional solid fats such as butter or shortening in various baked goods. These applications highlight the potential of oleogels to not only improve the nutritional profile of baked products but also to preserve or enhance their textural qualities and sensory appeal.

Table 2 emphasizes the significant role of oleogels in the baking sector. From Table 2, it can be found that cake is one of the main categories for oleogel application in bakery food. The desired porous crumb structure of a cake, marked by a well‐organized gas cell structure and high porosity, can be influenced by the incorporation of monoglyceride oleogel. Incorporating monoglyceride oleogel into cake formulations, it was observed that an increase in the oleogel proportion led to a decrease in the porosity values (Leila Roufegarinejad et al., 2023). Similar observations were made with highly binding oleogels (Chen et al., 2023; Meng et al., 2019), potentially due to oleogels' compact network structure increasing batter viscosity, which restricts bubble mobility. The hardness of cake is influenced by the type of oleogelator used, though a definitive conclusion has yet to be reached. For instance, Baştürk et al. (2023) observed a decrease in cake hardness as the ratio of wax increased, while Mert and Demirkesen (2016) reported the opposite phenomenon.

**TABLE 2:** Effect of oleogel applications on the texture and sensory properties of bakery products.

Food matrix	Oleogelator	Oil medium	Texture impacts	Sensory impacts	References
Cake	Emulsion templated	Sunflower oil	Light color; higher specific volume	—	Santos et al. (2024)
	Monoglyceride (7%)	Linseed oil, Sunflower oil	Comparable specific volume and color; Reduced porosity value	Reduced odor, color, and texture score	Roufegarinejad et al. (2023)
	Carnauba wax (6%)	Sunflower‐linseed oils	Lower porosity and specific volume	Odor and taste scores decreased	Roufegarinejad et al. (2024)
	Beeswax, rice bran wax (10%)	Sardine fish oil	Increased hardness with RBW oleogel, decreased hardness with BW oleogel; Larger pore size with RBW oleogel.	—	Ramadhan et al. (2023)
	Ethylcellulose/monoglyceride	Peanut diacylglycerol oil	—	50% substitution acceptable	Chen et al. (2023)
	Propolis wax (5%–10%), carnauba wax (4%–8%)	Safflower oil	—	Acceptance with carnauba wax oleogel	Baştürk et al. (2023)
	Monoglycerides (4%, 7%, 10%)	High oleic sunflower oil	Similar hardness, more homogeneous crumb structure	—	Giacomozzi et al. (2022)
Cookie	Ethylcellulose	Medium chain triglycerides	Softer texture	Higher acceptance	Jadhav et al. (2022)
Croissant	Hydroxypropyl methylcellulose	Sunflower oil	Chewier and more cohesive	50% substitution comparable with control	Espert et al. (2023)

The structured nature of oleogels contributes to improved flavor retention and distribution, resulting in a more pronounced and well‐rounded taste. This, in turn, enhances the sensory experience of baked goods. For instance, cookies made with oleogels exhibit a softer texture and reduced greasiness compared to those made with solid fats. A study by Yilmaz and Ogutcu (2015) discovered that cookies with sunflower wax and beeswax oleogels matched or surpassed the sensory attributes of shortening‐made cookies, according to a panel of 12. This improvement may be attributed to significant air entrapment during creaming and dough making, contributing to the desired cookie hardness. Similarly, Hwang et al. (2016) found no significant difference in texture between cookies made with various wax‐oil oleogels and those with commercial margarine, attributing this to comparable solid fat content and β’ crystal content, which are crucial for baking quality (Li et al., 2021). However, the impact of the oil matrix on texture can vary by baked item. Zheng et al. (2023) observed that diacylglycerol–ethylcellulose oleogels exhibited higher firmness compared to triacylglycerol–ethylcellulose oleogels, which could potentially result in increased hardness in sponge cakes and negatively impact consumer acceptance. To address this issue, emulsifiers such as monoglycerides can be utilized to enhance the softness of bakery products. Rodriguez‐Hernandez et al. (2021) suggested that emulsifiers like monoglyceride can efficiently incorporate more air bubbles into batters during mixing, mitigating the adverse effects of oleogels. However, it should be noted that different emulsifiers may operate through distinct mechanisms. Research by Chen et al. (2023) indicated that monoglyceride induced van der Waals forces in polar diacylglycerol oils, while hydrogen bond interactions occurred in triacylglycerol‐based oleogels.

An intriguing case study involving puff pastry was reported by Espert et al. (2023), who employed a hydroxypropyl methylcellulose (HPEC)‐structured sunflower oil oleogel as a fat alternative to enhance the nutritional profile of croissants. The fat used in puff pastry must have certain specific structural characteristics, such as predetermined plasticity, firmness, and solid fat content profile. During baking, the fat in the layer structure of laminated dough products causes each dough layer to bake separately, creating the characteristic visual separation of the layers and the flaky texture (Mattice & Marangoni, 2017). Their study demonstrated that the substitution led to croissants with lower saturated fat content and bite firmness, yet with a texture profile akin to those made with traditional shortening. The study observed that up to 100% oleogel presence did not detrimentally affect the firmness or springiness of croissants, though they became chewier and more cohesive with increased oleogel content.

### 3.3 Dairy products

As shown in Table 3, the exploration of oleogel applications in dairy products has been the focus of numerous studies, covering a range of products such as cheese (Huang et al., 2018), margarine (Wang et al., 2021), cream cheese (Bemer et al., 2016), ice cream (Airoldi et al., 2022), yogurt (Hyatt et al., 2023), among others. These investigations reveal that integrating oleogels into dairy formulations can markedly improve sensory qualities, including enhanced richness, smoothness, and overall mouthfeel. For instance, in a detailed sensory analysis conducted by da Silva et al. (2018) margarine variants containing an oleogel made from high‐oleic sunflower oil, candelilla wax, and monoacylglycerols were assessed by 120 panelists. Results indicated that the purchase intent for oleogel‐infused margarines was comparable to that for traditional commercial margarine. In the case of cheese production, incorporating oleogels has demonstrated benefits in texture, such as increased firmness, elasticity, and meltability (Huang et al., 2018). However, it is crucial to recognize that the hardness of processed cheese is influenced by the specific type and concentration of wax utilized. Airoldi et al. (2022) observed that while a sample with 50% carnauba wax oleogel matched the control in all tested parameters, a 100% carnauba wax oleogel sample, and others received lower acceptance. This diminished preference may be linked to the waxy aftertaste imparted by the oleogel, rendering the product less desirable for consumption (Garti & McClements, 2012). Furthermore, the application of oleogelators in yogurt has shown promise in reducing whey separation, enhancing the consistency and consumer appeal of the product (Hyatt et al., 2023).

**TABLE 3:** Effect of oleogel applications on the texture and sensory properties of dairy products.

Food matrix	Oleogelator	Oil medium	Texture impacts	Sensory impacts	References
Margarine	Beeswax, hydrocarbon (3%)	Sunflower oil	No significant color change, Decreased texture properties and melting enthalpy	No more than 30% oleogel replacement is acceptable; reduced consistency when oleogel replacement is higher than 40%	Sobolev et al. (2023)
	Monoglyceride stearate (12%)	Corn oil	Similar springiness, cohesiveness, gumminess, appearance, texture	Overall impression of commercial butter	Wang et al. (2021)
Yogurt	Monolaurin (12%)	Algal oil	Reduced “whey‐off”	—	Hyatt et al. (2023)
Ice cream	Carnauba wax (6%, 8%, 10%)	Soybean oil, peanut oil	Reduced melting rate, negatively affected overrun	No sensory impairment with 50% replacement of 6% oleogel	Airoldi et al. (2022)
Cheese	Rice bran wax, sunflower wax (0.5%, 1%)	Vegetable oil	Rice bran wax samples were harder than sunflower wax samples; the hardness of 1% rice bran wax sample comparable to the control	—	Huang et al. (2018)
Cream cheese	Hydroxypropyl methylcellulose	Sunflower oil	Enhance spreadability, decrease mechanical strength, softer texture	Improved spreadability, flavor on biscuits, and overall assessment affected by the amount of oleogelator	Wang et al. (2024)
	Rice bran wax, ethylcellulose (10%)	High‐oleic soybean oil	Sample with rice bran wax oleogel had similar harness and spreadability and stickiness	—	Bemer et al. (2016)

### 3.4 Others

The applications of oleogel in other products are shown in Table 4. Oleogels are revolutionizing the food industry, particularly in reformulating spread products to bolster nutritional profiles without detracting from their sensory and textural appeal. The mechanical attributes of oleogels, like spreadability and firmness, are adjustable through the careful selection and concentration of oleogelators, presenting a dynamic approach to tailoring the textural qualities of spreadable products. Oleogels successfully emulate the rich, mouth‐coating sensation typically associated with high‐fat spreads, ensuring a delightful sensory experience. Sensory evaluations have shown that consumers rate spreads made with oleogels similar to traditional options, underscoring the capability of oleogels to align with consumer preferences (Bascuas et al., 2021). Moreover, oleogels serve as effective carriers for flavors and bioactive ingredients, slowly releasing these elements to potentially enrich the flavor and nutritional content of spreads. Their capacity to retain and distribute flavors without compromising the product's perceived naturalness highlights the innovative potential of oleogels in next‐generation spread development. Nevertheless, optimizing oleogelator selection and formulation to mitigate any undesirable sensory qualities, such as waxy or greasy textures, remains crucial. Consumer studies stress the need for a harmonious blend of health advantages and sensory satisfaction, as the latter significantly influence purchase decisions.

**TABLE 4:** Effect of oleogel applications on the texture and sensory properties of other products.

Food matrix	Oleogelator	Oil medium	Texture impacts	Sensory impacts	References
Hazelnut cocoa spreads	Glycerol monostearate (3%, 6%)	Olive oil	Decreased spreadability, hardness	—	Marra et al. (2023)
Chocolate spread	Hydroxypropylmethylcellulose and xanthan gum	Olive oil, sunflower oil	Comparable spreadability with 50% replacement	Comparable “creamy appearance”, “creamy texture”, and “cocoa flavor” with 50% replacement	Bascuas et al. (2021)
Peanut butter	Beeswax, candelilla wax, rice bran wax, sunflower wax (1%–2%)	Peanut butter base	Wax types and the amount of wax had significant effects on the appearance, texture, and mouthfeel	Significant effect of wax type on sweetness and bitterness	Winkler‐Moser et al. (2022)
Chocolate	Monoglyceride stearate (3%)	Macadamia oil	No major impact on the mouthfeel; reduced hardness; increased yield stress and plastic viscosity	—	Espert et al. (2021)
Chicken nugget coating	Carnauba wax (5%)	Canola oil	Improved color; no significant difference in texture	Improved acceptances	Oyom et al. (2024)
Frying medium	Carnauba wax (5%, 10%, 15%)	Soybean oil	Similar crispiness	Overall, not as good as the control but 10% received the highest score	Chauhan et al. (2022)

In chocolate production, oleogels introduce a pioneering method to lessen dependency on cocoa butter and other saturated fats, offering healthier yet indulgent alternatives. The ability of oleogels to mimic the melting behavior of traditional fats ensures that the sensory experience remains intact, providing a smooth and velvety texture that is crucial for chocolate (Espert et al., 2021). The solid fat content (SFC) is a critical determinant of chocolate quality, with studies indicating a direct relationship between chocolate hardness and SFC (Kadivar et al., 2016). Recent findings suggest that oleogels can reduce the hardness of chocolate (Espert et al., 2021; Shuai et al., 2023), potentially altering the textural profile without significantly impacting the mouthfeel. This effect may be attributed to the lower saturated fatty acid content in oleogels compared to cocoa butter, resulting in a less robust crystal network. Moreover, the versatility of oleogels extends to chocolate spreads, enhancing their functionality. Spreadability assessments have shown that oleogels formulated with lower concentrations of stearic acid exhibit superior spreadability due to their increased elasticity (Sagiri et al., 2015).

As shown in Table 4, oleogels have also shown promise as frying mediums, offering a means to decrease oil absorption in fried foods and snacks, thereby potentially boosting sensory acceptance. Studies highlight a notable decrease in oil uptake when employing oleogels for frying, such as a 50% reduction in oil absorption in chicken breast fried in oleogel compared to traditional canola oil (Adrah et al., 2021). Similarly, frying potato chips in soybean oil structured with a carnauba wax oleogel resulted in 23% less oil absorption than chips fried in canola oil, with the oleogel‐fried chips also displaying a more appealing lightness (Thakur et al., 2023). The use of a soybean oil carnauba wax oleogel in preparing Mathri, a classic deep‐fried Indian snack, led to a 27% reduction in oil content, showcasing oleogels' effectiveness in minimizing oil absorption in fried foods (Chauhan et al., 2022). These outcomes emphasize the capacity of oleogels to enhance the sensory qualities and healthfulness of fried products without sacrificing their flavor or texture. As a versatile innovation, oleogels are proving to be instrumental in improving both the sensory experience and nutritional value of a wide range of culinary creations.

## 4 CHALLENGES AND LIMITATIONS OF OLEOGELS IN FOOD FORMULATIONS

Despite the potential of oleogels to enhance the texture and sensory attributes of food products, several challenges and limitations must be addressed to fully leverage their benefits in food formulations. A primary challenge lies in ensuring the compatibility of oleogels with various food matrices. While substituting saturated fats with oleogels can yield a healthier fatty acid profile, complete replacement often leads to decreased sensory acceptance due to adverse effects on texture and odor. For instance, wax‐based oleogels at higher concentrations can impart a waxy mouthfeel, and off‐flavors or odors can limit their applicability, especially in meat and bakery products. As evidenced in Tables 1, 2, 3, 4, partial replacement of saturated fats tends to achieve greater acceptability.

Moreover, maintaining the stability and consistency of oleogels under diverse storage and processing conditions is vital for their successful integration into food products. Temperature fluctuations, mechanical stress during processing, and prolonged storage can compromise the oleogel structure, causing oil syneresis, phase separation, or texture alterations. Developing oleogels with enhanced stability to withstand such challenges is paramount for their commercial success. Additionally, the use of oleogels in food products faces regulatory scrutiny, particularly regarding the safety and acceptability of oleogelators. Although the discovery of natural oleogelators is increasing, establishing regulatory guidelines for their usage in oleogel or fat‐substitute foods remains a challenge. Regulatory constraints on certain oleogelators could limit their application in food formulations. Ensuring compliance with food safety standards and securing approval from relevant authorities are critical hurdles for the widespread adoption of oleogel products.

The production of oleogels, especially those involving novel or specialized oleogelators, may incur higher costs compared to traditional fat‐based formulations. The expenses associated with procuring oleogelators, coupled with the necessity for modified processing equipment or methods, may elevate production costs. Achieving scalability while ensuring cost‐effectiveness is essential for the practical implementation of oleogels in the food industry.

## 5 CONCLUSION

The influence of oleogels on the texture and sensory characteristics of food products is complex and varies significantly across different types of food and their specific formulations. To optimize the desired texture and sensory outcomes, the selection of gelators and their dispersion methodologies must be carefully considered within the context of each food formulation. In addition to the discussed aspects of texture and sensory qualities, the stability of food products incorporating oleogels warrants attention, as the prevalent methods for oleogel preparation often involve blending an oleogelator with liquid oil under conditions of continuous stirring at elevated temperatures. Such conditions may adversely affect lipid oxidation, contributing to variable outcomes in oxidative stability. This variability is dictated by several factors, including the nature and amount of the structuring agent, the oil type, moisture content, and specific processing conditions.

It is feasible to formulate foods that mirror the textural qualities traditionally associated with solid fats. This enhancement in food functionality and quality is the result of precise control over oleogel manipulation. It is critical to acknowledge that the effective incorporation of oleogels into food products hinges on meticulous formulation and fine‐tuning of oleogel properties, including gelator selection, concentration, and processing parameters, which should be customized for each specific food formulation to meet desired texture and sensory standards. By thoughtfully addressing these intricacies, researchers and manufacturers have the opportunity to unlock the extensive promise of oleogels in the food sector, thereby creating pathways for the development of products that not only boast improved nutritional profiles but also deliver gratifying sensory experiences.

## AUTHOR CONTRIBUTIONS

**Lingyi Liu:** Writing – original draft (lead); methodology (equal); writing – review and editing (equal). **Zengli Gao:** Conceptualization (equal); writing – review and editing (equal). **Gang Chen:** Resources (supporting). **Jiaying Yao:** Software (supporting). **Xinyu Zhang:** Visualization (supporting). **Xiaoting Qiu:** Visualization (supporting). **Lianliang Liu:** Supervision (lead); methodology (supporting); writing – original draft (lead); writing – review and editing (equal).

## CONFLICT OF INTEREST STATEMENT

There is no conflict of interest between authors.

## Data Availability

Data sharing does not apply to this article as no datasets were generated or analyzed during the current study.
